# Prevalence and associated factors of hypertension among type 2 diabetes mellitus patients in Lautech teaching hospital, Osogbo, Nigeria

**DOI:** 10.4314/ahs.v23i4.34

**Published:** 2023-12

**Authors:** Opeyemi A Adeniyi, Olanrewaju D Eniade, Abayomi T Olarinmoye, Bukola A Abiodun, Omowumi O Okedare, Adenike A Eniade, James E Atolagbe

**Affiliations:** 1 Department of Public Health, Adeleke University, Ede, Osun State; 2 Department of Epidemiology and Medical Statistics University of Ibadan, Oyo State Nigeria; 3 Institute of Human Virology, Abuja Nigeria

**Keywords:** Type-2 Diabetes Mellitus, hypertension, older age, marriage, diet, alcohol intake

## Abstract

**Background:**

We assessed the prevalence and risk factors of hypertension among type-2 Diabetes Mellitus (DM) patients attending Ladoke Akintola University of Technology (LAUTECH) Teaching Hospital, Osogbo, Osun State Nigeria.

**Methods and materials:**

A hospital-based retrospective study was conducted among 143 type-2 DM patients in LAUTECH Teaching Hospital. Hypertension was defined as systolic BP ≥140 and or diastolic BP ≥90. Data were analysed using descriptive statistics, chi-square, and binary logistic regression.

**Results:**

The mean age of the respondents was 56.2 ±15.79 years. Hypertension was common (32.1%) among type-2 DM participants. Respondents aged 45-64 years (OR= 5.96, 95%CI= 1.60 – 19.12) had the likelihood of being hypertensive. Type-2 DM patients who were not in union (AOR=6.64, 95%CI=1.79 – 24.52) were more likely to be hypertensive. The likelihood of hypertension was lower (AOR= 0.28, 95%CI=0.11 – 0.66) among participants who engaged in moderate physical activity compared to those who engaged in low physical activity.

**Conclusion:**

This study identified the age group 45-64 years, not being in a union and engagement in low physical activity as associated factors for hypertension among Diabetes Mellitus participants. Hypertension prevention/treatment should be considered in type-2 Diabetes Mellitus routine treatment.

## Background

Hypertension is a chronic disease characterized by persistently raised blood pressure. It is a multi-factorial disorder that may be precipitated in genetically susceptible individuals in the presence of the right environmental risk factors. (Ahmed *et.al*; 2015). Blood pressure is determined from the systolic and diastolic numbers. The systolic measures the pressure in blood vessels when the heart contracts, while the diastolic measures the pressure in the blood vessels when the heart relaxes (i.e. the pressure in between the heartbeats) (WHO, 2021). High blood pressure (hypertension) is diagnosed as ≥140 mmHg systolic blood pressure reading and/or ≥90 mmHg diastolic blood pressure reading measured twice on different days (WHO, 2021).

According to the World Health Organization, about 1.28 billion adults (aged 20-79 years) globally have raised blood pressure. Hypertension has been identified as a leading cause of untimely death worldwide (WHO, 2021). About 66.7% of the global burden of hypertension is in low-middle-income countries and a high (30.6%) prevalence of hypertension has been reported in Nigeria (WHO, 2021; Adeloye et al., 2021).

Diabetes Mellitus (DM) is a chronic metabolic condition that is typified by persistently raised blood glucose levels (WHO, 2006). Diabetes occurs when the body does not produce enough insulin (the hormone that regulates blood sugar) or the body cannot effectively use the insulin produced. As medically referred to, hyperglycemia/raised blood sugar causes serious damage to body systems such as nerves and blood vessels (World Health Organization, 2021a). In 2019, diabetes was identified as the ninth leading cause of death globally. It was responsible for an estimated 1.5 million mortality (World Health Organization, 2021a). The Centre for Disease Control described the types of diabetes as type-1 and type-2. Type-1 diabetes is caused by an autoimmune reaction which stops the body from making insulin. It has no known prevention and accounts for about 5 – 10% of diabetes types. Type-1 diabetes is common among children, teens, and young adults (CDC, 2022). Type-2 diabetes occurs when the body is not able to maintain a normal blood sugar level due to the inability to properly use insulin. Type-2 diabetes is common among adults and it can be prevented or delayed with a healthy lifestyle (CDC, 2022). Research has documented that the prevalence of hypertension is higher among diabetic patients compared to the non-diabetic population. The co-existence of hypertension and type 2 DM elevates the risk of heart diseases, nephropathy, stroke and retinopathy-micro-vascular and macro-vascular complications. (Berraho *et.al*; 2012).

Studies have revealed that hypertension contributes to the development of long-term complications in type 2 Diabetes Mellitus. Hypertension and obesity also, separately, or together, are common co-morbidities in adults with type-2 DM (Colosia *et.al*; 2013). The co-existence of these two health conditions is more harmful to the populations in low-income countries than in high-income countries due to poor access to healthcare and inadequate preventive programmes. (Colosia *et.al*; 2013). According to Unadike *et.al*; 2011, in a study to determine the prevalence of hypertension among people with Diabetes Mellitus in Benin City, 54.2% of the respondents with DM were hypertensive on clinical examination. Another study conducted in Morocco revealed a prevalence of 75% among type-2 Diabetes mellitus patients. The same study found a significant association between hypertension and older age, Body Mass Index and duration of DM. It also revealed that overweight and obese type 2 Diabetes Mellitus patients have a higher risk of hypertension than those with normal BMI, though there was no significant association with smoking in the participating patients (Berraho *et.al*; 2012).

The American Diabetes Mellitus Association reported that knowledge and management of the risk factors for hypertension in type-2 DM patients will be helpful to determine the resources needed to reduce the burden of the risk factors and prevent and/or manage cardiovascular complications (Colosia et.al; 2013). High prevalence of hypertension (45.6%) and DM (23.3%) have been reported in southwest Nieria (Bello-Ovosi *et al.*, 2018; Opreh *et al.*, 2021). Another peculiarity in southwest Nigeria is the unhealthy pattern of predisposing nutrient intake, nutritional status, and lifestyle (Afolabi *et al.*, 2015). However, there is a paucity of information on the episode of hypertension and type-2 DM in this southwest region of Nigeria. Carrying out this study in this population will provide adequate information on the prevalence of hypertension and its associated factors among the type-2 DM patient in this population. Hence, this study aimed to assess the prevalence and associated factors of hypertension in the type 2 Diabetes Mellitus patients attending the Ladoke Akintola University of Technology (LAUTECH) Teaching Hospital, Osogbo, Osun-State Nigeria.

## Methods and materials

### Study design

A retrospective assessment of type-2 Diabetes Mellitus patients in the Ladoke Akintola University of Technology Teaching Hospital (LAUTECH Teaching Hospital).

### Study population

The LAUTECH Teaching Hospital is now known as the University of Osun State Teaching Hospital (UNIOSUN Teaching Hospital). The hospital is located in Osogbo, Osun State, Nigeria to provide tertiary health care and support undergraduate medical students from LAUTECH. The clinic has an average attendance of 2000 clients on regular follow-ups and more than 150 patients attending the diabetes clinic every month.

### Sampling technique

We carried out a retrospective study using secondary data of type-2 Diabetes Mellitus patients attending the DM clinic of the LAUTECH Teaching Hospital, Osun state, Nigeria. Data were gathered over three years from January 1, 2017, to December 31, 2019. A simple random sampling method was employed to select the hospital identity numbers of the type-2 DM patients from the clinic records. We then extracted information from the files of the participants. After being selected, patients were invited to participate further in the study to provide information on their lifestyle and dietary pattern.

### Inclusion Criteria

Type-2 DM patients who presented at the Diabetes Mellitus clinic within the time frame of this study were included in this study.

### Exclusion Criteria

Type-2 Diabetes Mellitus patients who were severely ill and could not participate were excluded from the study.

### Variables

Outcome variable hypertension was defined by the WHO. as systolic BP ≥140mmHg and or diastolic BP ≥90mmHg), coded as Yes = 1 for hypertensive participants and No = 0 for non-hypertensive participants. Variables such as demographics, systolic and diastolic blood pressure, lipid profile, and blood glucose level were extracted from the records of the patients. Also, a structured data collection form was used to elicit more information from the patients. Information on lifestyles such as level of physical activities, alcohol intake, and use of tobacco was considered as independent variables. The physical activity was categorized as low, moderate, and high using the based on International physical activity questionnaire-short form (IPAQ) (Craig et al., 2017). In addition, information on patients' weight and height was obtained from the patient's records. Weight and height were used to classify participants' body mass index (BMI) into normal (18.5 – 24.9kg/m2), overweight (25 – 29.9kg/m2), and obese (≥ 30kg/m2). The information on fat and oil, fibre, and carbohydrate was obtained using the Food Frequency Questionnaire (FFQ) and the scoring guide was used to determine the categories (Cade, Burley, Warm, Thompson, & Margetts, 2004).

### Data analysis

The data collected were entered into the IBM-Statistical package for social sciences (IBM-SPSS) version 25 (I B M SPSS, 2017) and analysed using Stata version 16 (Stata corps, 2018). Also, frequency and percentage distribution were generated to describe participants' demographic characteristics, lifestyle and dietary patterns of type 2 DM patients and determine the prevalence of hypertension. Also, mean and standard deviations were calculated to summarize quantitative variables such as age, systolic blood pressure and diastolic blood pressure. The Chi-square test was used to investigate associations between hypertension and the patient's profile. A binary logistic regression model was used to investigate the influence of lifestyle, dietary patterns and demographic characteristics on hypertension among type-2 Dm patients. Variables were considered significant if the p-value was less than 5%.

### Ethical approval

Approval for the study was obtained from the Adeleke University Ethical Review Committee (AUREC), Ede, State of Osun (Reference number: AUERC/FBMS/01) and the Research and Ethics Committee of LAUTECH Teaching Hospital, Osogbo (Protocol number: LTH/EC/2020/06/459). Also, confidentiality and anonymity of the patients' data were ensured by identifying patients by their hospital numbers and data collection forms were kept in a locked cabinet and entered into a password-protected computer only accessible to the biostatistician in the team.

## Results

### Profile, lifestyle and dietary pattern of the participants

**Figure 1:**
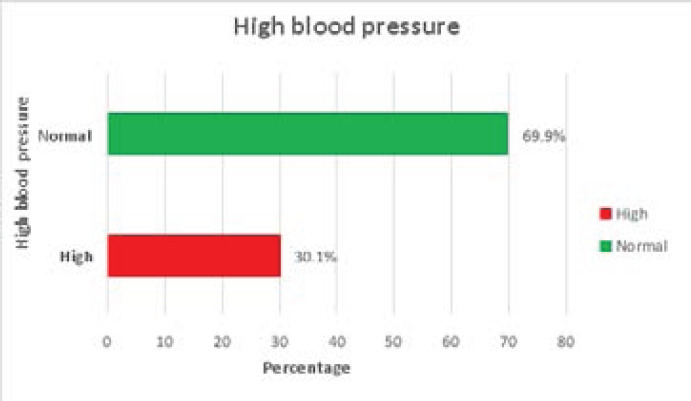
Prevalence of hypertension among type-2 DM patients

The profile, lifestyle and dietary patterns of the study participants were presented in table 1 and figure 1 below. The mean age of the respondents was 56.2 ±15.79 years. About 42% and 32% of the participants were middle-aged adults and older adults respectively. More females (60.8%), currently working class (61.5%) and Christians (67.8%) participated in this study. Less than one-tenth (8.4%) of participants engaged in a high level of physical activity. The majority reported a high level of carbohydrate intake (76.8%), high fats and oil intake (52.8%) and fibre intake (54.3%) were recorded among the participants. Animal fat was the common (65.7%) source of fat consumed by the respondents. Few of the participants smoke (7.7%) and drink alcohol (19.3%). The prevalence of hypertension was high (32.1%) among the study participants

**Table 1:** Profile, lifestyle and dietary pattern of type-2 DM patients

Variables	Frequency (n=143)	Percentage (%)
**Age in years** Mean (SD)	56.2(15.79)	
**Age group**		
18 -44 (young adults)	37	25.9
45 -64 (Middle-aged adults)	60	42.0
>=65 (Older adults)	46	32.2
**Gender**		
Male	56	39.2
Female	87	60.8
**Work status**		
Currently working	88	61.5
Not currently working	55	38.5
**Marital status**		
Currently in a Union	127	88.8
Not currently in a union	16	11.2
**Religion**		
Islam	46	32.2
Christianity	97	67.8
**Level of education**		
None	15	10.5
Primary	28	19.6
Secondary	31	21.7
Tertiary	69	48.3
**Tribe of respondents**		
Yoruba	137	95.8
Other (Igbo & Hausa)	6	4.2
**Level of physical Activity**		
Low	63	44.1
Moderate	68	47.6
High	12	8.4
* **Carbohydrate (n=142)**		
Low	33	23.2
High	109	76.8
* **Fats and Oil (n=142)**		
Low	67	47.2
High	75	52.8
* **Fibre (n=140)**		
Low	64	45.7
High	76	54.3
**Source of fat**		
Animal source	94	65.7
Plant source	49	34.3
**Smoke**		
Yes	11	7.7
No	132	92.3
* **Alcohol intake (n=140)**		
Yes	27	19.3
No	113	80.7

* **Missing data**

### Association between hypertension and profile, lifestyle and dietary pattern of the participants

Table 2 showed the association between hypertension and the profile of participants who have type-2 DM. Hypertension was more pronounced (38.3%) among the participants aged 45-64 years old (P=0.032). Similarly, cases of hypertension were more common (50%) among participants who were not in a union compared to those (27.6%) who were in a union (P=0.065). The majority (48.0%) of hypertensive participants had raised total cholesterol compared to the 26.3% who had normal total cholesterol levels (P=0.031). Two-fifth of the hypertension patients (42.9%) engaged in low physical activities(P=0.012).

**Table 2:** Association between Hypertension and Profile, lifestyle and dietary pattern of the participants

	High blood pressure (n=143)		Chi-square	P value
Variables	Normal	High blood pressure		
**Age**			6.91	0.032
18 -44 (young adults)	32(86.5)	5(13.5)		
45 -64yrs (Middle-aged adults)	37(61.7)	23(38.3)		
>=65 yrs. (Older adults)	31(67.4)	15(32.6)		
**Occupation**			0.30	0.584
Currently working	63(71.6)	25(28.4)		
Not currently working	37(67.3)	18(32.7)		
**Marital status**			3.40	0.065
Currently in a Union	92(72.4)	35(27.6)		
Not currently in a union	8(50.0)	8(50.0)		
**Level of education**			0.90	0.824
None	12(80.0)	3(20.0)		
Primary	19(67.9)	9(32.1)		
Secondary	22(71.0)	9(29.0)		
Tertiary	47(68.1)	22(31.9)		
**Tribe of respondents**			0.69	0.711
Yoruba	96(70.1)	41(29.9)		
Others (Igbo, Hausa)	4(66.7)	2(33.3)		
**Religion**			0.51	0.474
Islam	34(73.9)	12(26.1)		
Christianity	66(68.0)	31(32.0)		
**Gender**			2.06	0.151
Male	43(76.8)	13(23.2)		
Female	57(65.5)	30(34.5)		
**Total cholesterol**			4.63	0.031
Normal	87(73.7)	31(26.3)		
Raised	13(52.0)	12(48.0)		
**HDL**			0.21	0.647
Reduced	22(73.3)	8(26.7)		
Normal	78(69.0)	35(31.0)		
**LDL**			2.57	0.109
Normal	97(71.3)	39(28.7)		
Raised	3(42.9)	4(57.1)		
**Alcohol intake**			3.38	0.066
Yes	23(85.2)	4(14.8)		
No	76(67.3)	37(32.7)		
**Smoke**			0.04	0.833
Yes	8(72.7)	3(27.3)		
No	92(69.7)	40(30.3)		
**Source of fat consumed by the participants**			2.06	0.151
Animal source	62(66.0)	32(34.0)		
Plant source	38(77.6)	11(22.4)		
**Dietary pattern: Fibre**			0.44	0.505
Low	43(67.2)	21(32.8)		
High	55(72.4)	21(27.6)		
**Dietary pattern: Fats and Oil**			0.45	0.503
Low	49(73.1)	18(26.9)		
High	51(68.0)	24(32.0)		
**Dietary pattern: Carbohydrate**			1.68	0.196
Low	26(78.8)	7(21.2)		
High	73(67.0)	36(33.0)		
**Level of engagement in physical activity**			8.83	0.012
Low	36(57.1)	27(42.9)		
Moderate	54(79.4)	14(20.6)		
High	10(83.3)	2(16.7)		
**BMI (Kg/m2)**			4.49	0.106
Normal	42(76.4)	13(23.6)		
Overweight	30(58.8)	21(41.2)		
Obese	27(75.0)	9(25.0)		

### Associated factors of hypertension among type-2 DM patients in LAUTECH Teaching Hospital

The result of the bivariate and multivariate analysis are presented in table 3. We found that age was a determinant of hypertension among type-2 DM patients, respondents aged 45-64 years (OR= 5.69, 95%CI= 1.60 – 19.12) were more likely to be hypertensive compared to the age group 18 -44 years. Also, type-2 DM participants who were not in a union (AOR=6.64, 95%CI=1.79 – 24.52) were more likely to be hypertensive compared to those who were in a union. This study also revealed that the likelihood of hypertension was lower (AOR= 0.28, 95%CI=0.11 – 0.66) among participants who were engaged in moderate physical activity relative to those who were engaged in low physical activity. Comparing the estimates in the bivariate analysis to the multivariate analysis, age and marital status were identified as confounding variables in the results.

**Table 3:** Risk factors of hypertension among type-2 DM patients in LAUTECH Teaching Hospital

	Unadjusted Odds Ratio				Adjusted Odds Ratio			
			95% CI				95% CI	
	OR	P Value	Lower	Upper	AOR	P Value	Lower	Upper
**Variables**								
**Age**								
18 -44 (young adults)	reference				reference			
45 -64yrs (Middle-aged adults)	3.98	0.012*	1.36	11.68	5.69	0.005**	1.69	19.12
>=65 years (Older adults (Elderly))	3.10	0.049	1.00	9.55	2.28	0.191	0.66	7.84
**Marital status**								
Currently in a Union	reference				reference			
Not currently in a union	2.63	0.072	0.92	7.54	6.64	0.005**	1.79	24.52
**Total cholesterol**								
Normal	reference				reference			
Raised	2.59	0.035*	1.07	6.28	1.88	0.203	0.71	4.97
**Level of engagement in physical Activity**								
Low	reference				reference			
Moderate	0.35	0.007*	0.16	0.75	0.28	0.004**	0.11	0.66
High	0.27	0.105	0.05	1.32	0.47	0.411	0.08	2.81

* *p significant for unadjusted odds ratio*

** *p significant for adjusted odds ratio*

## Discussion

This study showed that hypertension is a common co-morbidity among type 2 DM patients attending LAUTECH Teaching Hospital, Ogbomoso. About one-third of type-2 DM participants had high blood pressure. The prevalence of hypertension among DM patients in our study is consistent with other studies (Chowdhury *et al.*, 2021), but lower than studies in Cameroon (Kemche et al., 2020), Benin Republic (Amoussou-guenou *et al.*, 2015), Ethiopia (Akalu & Belsti, 2020; Tadesse *et al.*, 2018), and Morocco (Berraho *et al.*, 2012). The difference could be attributed to the difference in the participants' age. Our participants were younger than those studies that reported high prevalence. Consistent with other studies (Abdullah & Mansour, 2020; Amoussou-guenou et al., 2015; Tadesse *et al.*, 2018), increased age was associated with hypertension among type 2 DM participants. Increasing age has been established as a risk factor for hypertension, and type 2 DM. A possible explanation is that older people engage less in physical activity capable of reducing body weight. It has been documented that ageing reduces the elasticity of the blood vessels and increases the rigidity of the vessels, thereby elevating blood pressure (Rizzoni *et al.*, 2019; Zhou *et al.*, 2014). We found that raised cholesterol increased the risk of hypertension among our respondents by almost twice. Raised cholesterol could occur because of high BMI and lack of participation in physical activity (Amoussou-guenou *et al.*, 2015; Kemche *et al.*, 2020) which was also documented in this study. The majority of our respondents reported intake of high carbohydrate, and fats & oil diets. These are capable of increasing body weight, coupled with a lack of moderate -high level of physical activity (Van Dam & Seidell, 2007).

We found that participants who were not currently in a union had a higher risk of hypertension. This finding is in line with the literature, studies have identified an association between marital status and the risk of hypertension, DM and mortality (Ramezankhani *et al.*, 2019). Although there is no existing theory or mechanism to explain the influence of marital status on the risk of hypertension, we could link this to the fact that having a spouse or children may increase the likelihood of engaging in physical activity or consciousness of one's health.

## Strength

We reported a notable prevalence of hypertension among type-2 DM patients and we also documented the associated factors of hypertension among type-2 diabetes mellitus patients in the southwest region, of Nigeria, a population identified for an unhealthy pattern of predisposing nutrient intake, nutritional status, and lifestyle.

## Limitation

Although we could not establish causality, it is clear from our findings that both diseases are interrelated. Another limitation of our study is that we did not explore the duration of DM among the patients and could not document the number of participants who are on anti-hypertensive.

## Conclusion

This study identified the age group 45-64 years, not being in a union and engagement in low physical activity as associated factors for hypertension among Diabetes Mellitus participants. We corroborate routine screening as a prevention strategy for hypertension among type-2 DM patients. Similarly, type-2 DM patients should be encouraged to engage in moderate physical activity. Hospital management can also provide exercise opportunities for all patients during their clinic visits. Health education focused on lifestyle modification will be helpful to create awareness among the general population.
